# Evaluating the Usability and Perceived Impact of an Electronic Medical Record Toolkit for Atrial Fibrillation Management in Primary Care: A Mixed-Methods Study Incorporating Human Factors Design

**DOI:** 10.2196/humanfactors.4289

**Published:** 2016-02-17

**Authors:** Kim Tran, Kori Leblanc, Alissia Valentinis, Doug Kavanagh, Nina Zahr, Noah M Ivers

**Affiliations:** ^1^ OpenLab University Health Network Toronto, ON Canada; ^2^ Department of Pharmacy University Health Network Toronto, ON Canada; ^3^ Leslie Dan Faculty of Pharmacy University of Toronto Toronto, ON Canada; ^4^ Taddle Creek Family Health Team Toronto, ON Canada; ^5^ North York Family Health Team Toronto, ON Canada; ^6^ Women's College Hospital Department of Family and Community Medicine University of Toronto Toronto, ON Canada

**Keywords:** primary health care, atrial fibrillation, electronic health records, mixed-methods research, evidence-based medicine

## Abstract

**Background:**

Atrial fibrillation (AF) is a common and preventable cause of stroke. Barriers to reducing stroke risk through appropriate prescribing have been identified at the system, provider, and patient levels. To ensure a multifaceted initiative to address these barriers is effective, it is essential to incorporate user-centered design to ensure all intervention components are optimized for users.

**Objective:**

To test the usability of an electronic medical record (EMR) toolkit for AF in primary care with the goal of further refining the intervention to meet the needs of primary care clinicians.

**Methods:**

An EMR-based toolkit for AF was created and optimized through usability testing and iterative redesign incorporating a human factors approach. A mixed-methods pilot study consisting of observations, semi-structured interviews, and surveys was conducted to examine usability and perceived impact on patient care and workflow.

**Results:**

A total of 14 clinicians (13 family physicians and 1 nurse practitioner) participated in the study. Nine iterations of the toolkit were created in response to feedback from clinicians and the research team; interface-related changes were made, additional AF-related resources were added, and functionality issues were fixed to make the toolkit more effective. After improvements were made, clinicians expressed that the toolkit improved accessibility to AF-related information and resources, served as a reminder for guideline-concordant AF management, and was easy to use. Most clinicians intended to continue using the toolkit for patient care. With respect to impact on care, clinicians believed the toolkit increased the thoroughness of their assessments for patients with AF and improved the quality of AF-related care received by their patients.

**Conclusions:**

The positive feedback surrounding the EMR toolkit for AF and its perceived impact on patient care can be attributed to the adoption of a user-centered design that merged clinically important information about AF management with user needs. This study demonstrates the utility of a human factors approach to piloting and refining an intervention prior to wide-scale implementation.

## Introduction

Atrial fibrillation (AF) is a common and preventable cause of stroke [1]. The prevalence of AF is approximately 1% overall; however, it accounts for 15% of all strokes and 33% of all strokes in the elderly [2]. Such strokes result in permanent disability in 60% and death in 20% of individuals [3]. Medications can effectively reduce the risk of stroke. Unfortunately, although evidence has long been available that many AF-related strokes are preventable with proper therapy, the proportion of eligible patients receiving appropriate stroke prevention therapy remains stubbornly low. The 2012 Canadian Cardiovascular Society guidelines for AF emphasize that the vast majority of patients with AF would likely benefit from anticoagulation to reduce risk of stroke [4]. However, studies have found that many patients at high risk of stroke are not receiving anticoagulation.

Barriers to appropriate stroke prevention therapy may exist at the system, physician, and patient level. At the system level, primary care clinics were found to have inadequate coordination with laboratories, ineffective INR tracking systems, and inefficient use of reminders [5]. Physicians tend to overestimate the risk of bleeding associated with anticoagulation, especially in the elderly, even though guidelines state that the benefits of anticoagulation outweigh the risks for most patients over 65 years of age [6-8]. In contrast, patients were found to place more value on avoidance of stroke than avoidance of bleeding [9]. In the context of infrequent use of formal risk assessment tools and underutilization of anticoagulation, it is plausible that tools supporting evidence-informed, shared decision-making processes with patients may lead to increased utilization of anticoagulation [10,11].

Electronic medical record (EMR) interventions have been described as instrumental for chronic disease management. Useful aspects of these interventions include decision support such as reminders for patient care, clinical monitoring through large-scale surveillance and data aggregation, and disease-specific encounter templates [12,13]. These EMR-based interventions have been shown to improve quality of care, improve efficiency, and decrease health services utilization [12-14].

We developed an EMR-based toolkit of quality improvement strategies to aid atrial fibrillation management in primary care, to improve the proportion of patients receiving guideline-concordant stroke prevention therapy. The objective of this pilot study was to test the usability of the EMR toolkit for AF in primary care with the goal of further refining the intervention to meet the needs of primary care clinicians. A human factors approach was utilized in the development of the toolkit in an effort to optimize the uptake of the toolkit, which in turn, would have the potential to increase the proportion of patients receiving guideline-concordant AF care.

## Methods

### Study Design

We conducted a pilot study using a mixed-methods approach consisting of observations, semi-structured interviews, and surveys to examine the usability of an EMR toolkit for AF and its perceived impact on care and workflow. A human factors approach, defined as “the study of the interrelationship between humans, the tools and equipment they use in the workplace, and the environment in which they work” was used to optimize usability and uptake of the toolkit [15]. The study received approval from the research ethics board at the University of Toronto.

### Study Population

The study was conducted between October 2013 and July 2014. Participants were primary care clinicians at the Taddle Creek Family Health Team (TCFHT) and Women’s College Hospital (WCH) Family Practice Health Centre in Toronto, Ontario, Canada. The TCFHT consists of 14 family physicians, 3 nurse practitioners, 3 nurses, 3 social workers, 2 pharmacists, and a dietician who provide primary health care to 18,900 patients. The WCH Family Practice Health Centre consists of 31 family physicians, 2 nurse practitioners, 16 nurses, 2 dieticians, 1 occupational therapist, 2 social workers, and 1 pharmacist who provide primary health care to 18,500 patients.

### EMR Toolkit Development

The purpose of the EMR toolkit for AF, which links to PS Suite EMR, is to facilitate the uptake and translation of knowledge regarding guideline-based AF management in primary care and to improve the proportion of patients receiving guideline-based AF care. The toolkit was developed by an interdisciplinary team with clinical and design expertise. The clinical content was developed by 3 family physicians, a pharmacist, and a cardiologist with expertise in primary care and AF management. A designer provided human-centered design expertise and created mockups of the design and layout of the toolkit. Toolkit development occurred through an iterative process with iterations cycled back through the team prior to the commencement of formal usability testing. The EMR toolkit incorporated evidence-based recommendations for AF management specified in the 2012 Canadian Cardiovascular Society AF guidelines. The final version consisted of a toolbar embedded into the electronic medical charts of patients with AF that included the following tools: (1) initial assessment form; (2) follow-up visit form; (3) stroke and bleeding risk calculator, which included guideline-concordant recommendations for treatment; (4) provider resources; and (5) patient resources. The outputs from Tools 1 to 3 are embedded into patients’ electronic medical charts. The provider resources consisted of three documents: (1) AFib in One Page, (2) Comparison of Anticoagulants, and (3) Anticoagulation Dosing Table. The patient resources consisted of educational documents providing information on atrial fibrillation, what to do if a patient experiences an AF episode, treatment options, decreasing stroke risk, available anticoagulants, and cardioversion.

### Usability Testing

The EMR toolkit underwent usability testing using a qualitative approach consisting of observations of primary care clinicians’ interactions with the EMR toolkit for AF and semi-structured interviews with family physicians. A purposive sample of primary care clinicians from the Taddle Creek Family Health Team and Women’s College Hospital Family Practice Health Centre was selected for the study.

Usability testing was conducted by members of the research team (KT and KL) and occurred in two phases. Phase 1 used the “think aloud” approach while primary care clinicians used the toolkit with test patients. Field notes employing a structured data collection form were used to record usability-related issues. Phase 2 consisted of observations of primary care clinicians’ interactions with the toolkit as they conducted a visit with an actual patient with AF; observations were followed by semi-structured interviews to examine their perceptions of the toolkit. Field notes employing a structured data collection form were used to record usability-related issues during observations. Interviews were audiotaped and transcribed by a member of the research team (KT). Participant feedback was used iteratively to make improvements to the toolkit.

Inductive thematic analysis was conducted using qualitative data analysis software (NVivo 10, QSR International). Two members of the research team (KT and KL) independently coded 4 interview transcripts to identify interesting features of the data and provide a comprehensive selection of codes, which were reviewed and discussed to ensure consensus. A coding framework was developed using initial findings to guide coding of remaining interviews and field notes, with additional codes reported as they were identified. Initial codes and their coded interview extracts were organized into themes and subthemes. All themes and coded interview extracts were reviewed to ensure data within each theme formed a coherent pattern and a clear distinction between different themes was evident.

### Surveys

A survey was developed by the research team to examine primary care clinicians’ perceptions of the EMR toolkit for AF and its impact on patient care and management. A subset of questions was aligned with the key themes identified through usability testing. The research team reviewed the survey for face validity, comprehensiveness, and clarity. Pre-testing occurred with 2 individuals with research backgrounds who reviewed the survey for face validity and clarity. The final survey consisted of 22 questions with a combination of multiple choice, rating scale, and open-ended items. A 5-point Likert scale and 5-point scale were used to express level of agreement and degree of change, respectively.

The survey was distributed electronically to all primary care clinicians—family physicians, nurse practitioners, nurses, and pharmacists—at the TCFHT. An initial introduction email followed by 2 reminder emails was sent to all clinicians to provide them with information regarding the study and the electronic survey. To increase the response rate, paper surveys were also distributed to clinicians following the third email reminder. Descriptive statistics were generated from the survey data.

## Results

### Participants

A total of 14 clinicians (13 FPs and 1 NP) participated in Phase 1 or 2 of usability testing. Each clinician was observed as they used the EMR toolkit for AF with either a test patient (Phase 1-5 FPs and 1 NP) or a real patient with AF (Phase 2-8 FPs). Semi-structured interviews were conducted with all 8 FPs who participated in Phase 2 of the usability testing. The response rate for the survey was 55% (12/22). A total of 8 family physicians, 2 nurses, 1 nurse practitioner, and 1 pharmacist participated in the survey.

### Phase 1 Usability Testing: Key Themes

Participants highlighted several usability-related issues that required improvements. Three overarching themes were identified, which included (1) interface-related changes, (2) additional resources for AF management, and (3) toolkit functionality issues. In total, the EMR toolkit for AF underwent 9 iterative cycles of changes based on participants’ feedback to produce the final version.

Participants highlighted several interface-related issues concerning the EMR toolkit’s layout, format, and language. They described a lack of an intuitive flow for the layout of the initial assessment and follow-up visit forms, confusion surrounding the format of checkboxes, and confusion around the use of abbreviations. In response, we modified the layout, format, and language to make the toolkit more user-friendly. For the layout, we optimized the structure of the assessment forms to align with the SOAP (subjective, objective, assessment, and plan) note structure, which participants described as their typical workflow when writing chart notes. Improvements were made to the formatting by creating checkboxes with yes/no options rather than checkmarks, which participants felt was better for identifying if they had missed sections. Lastly, modifications to the language of the toolkit were made to improve clarity. For example, the abbreviation OAC was changed to oral anticoagulation.

Participants expressed a desire to have additional resources for AF management. In response, we created additional resources to aid clinician decision making. A “Provider Resources” section was added to the toolbar that provides clinicians with (1) AFib in One Page, a 1-page document that provides guideline-based recommendations for rate/rhythm control and stroke prevention management; (2) Comparison of Anticoagulants, a 1-page document comparing the effectiveness and safety of the available anticoagulants for stroke prevention; and (3) Anticoagulation Dosing Table, a 1-page document providing dosage recommendations for all of the available anticoagulants. Additionally, some participants were unclear on how to rate their patients’ AF using the Severity of AF (SAF) scale found on the initial assessment and follow-up visit forms. In response, a “learn more” button was added that provided definitions for each SAF class.

Lastly, participants described issues relating to the functionality of the EMR toolkit. For example, several links and buttons embedded in the toolkit did not work properly. In response, these functionality-related issues were fixed by our programmer.

### Phase 2 Usability Testing: Key Themes

The key themes identified from Phase 2 usability testing can be grouped into the following sections: features that would promote use of the EMR toolkit of AF, areas for improvement, and perceived impact of the toolkit on patient care and workflow. No major usability-related issues were identified in Phase 2 of usability testing.

#### Features That Would Promote Use of the EMR Toolkit for AF

Participants described several benefits to using the EMR toolkit for AF. Three main subthemes were identified: (1) ease of accessibility to AF-related information and resources is beneficial, (2) structured guide for AF management serves as a reminder, and (3) structure and format of the toolkit were easy to use and follow.

##### Ease of Accessibility to AF-Related Information and Resources is Beneficial

A common advantage of the EMR toolkit for AF described by users was the accessibility of AF-related information and resources provided to clinicians. Participants described that they liked the fact that information was available to them at the “touch of a button,” as information pertaining to AF management could be conveniently found in the Patient Resources, Provider Resources, and Stroke & Bleeding Risk Calculator sections of the toolkit. A family physician described, “there’s so much information and I love the provider resources as well because sometimes you think you’re not sure about something.” With respect to the Stroke & Bleeding Risk Calculator, one participant stated that she liked that “you can pull up the CHADS score easily so you don’t have to remember the whole thing if you’re not sure.” Another family physician described the time-saving benefit of using the toolkit and the lengthy process of accessing information without the toolkit:

I think it would save me time, it saves time to have it embedded. I mean we certainly do have patient handouts in the EMR but I’d have to come here and go (to) handouts and then look for atrial [fibrillation]...it would be I assume scanned in under atrial fibrillation and then click it and then view it and then print it.

From survey results, 91% (10/11) of participants believed the EMR toolkit for AF improved their ability to access the information they needed to provide AF care.

##### Structured Guide for AF Management Serves as a Reminder

Most participants felt that the structured guide for AF management served as a reminder for what to ask patients regarding their AF care. A family physician stated that the toolkit was “good because...like everything else it gives you a list [so] you don’t forget what you’re supposed to be check[ing] which especially on a busy day you tend to rush and miss stuff.” One family physician described how the toolkit served as a cheat sheet and ensured a systematic approach was taken towards the management of patients with AF:

I think it’s great, it is to me a real cheat sheet, like it makes sure you don’t miss anything and that you do go through an organized, systematic approach to dealing with atrial [fibrillation]...I just think it’s really efficient and it makes sure you...do what you’re supposed to do.

##### Structure and Format of the EMR Toolkit for AF Was Easy to Use and Follow

Participants felt that the EMR toolkit for AF was easy to use and follow. They described how the toolkit was clear, intuitive, and straightforward. A family physician stated “sometimes [with] forms you can’t find what you need to do,” but the EMR toolkit was “fine...it was very easy to use.” Another family physician described the intuitive nature of the initial assessment form:

It was actually pretty intuitive...In terms of obtaining a history of atrial fibrillation, so asking about symptoms, asking about risk factors and then examining the patient and coming up with their stroke risk and plan, that is very intuitive flow.

From survey results, 100% (11/11) of participants thought that aspects of the EMR toolkit for AF were easy to use. Most (82%, 9/11) participants felt that the EMR toolkit for AF was compatible with their typical workflow.

#### Areas for Improvement

Participants suggested changes that could be made to improve the toolkit and its uptake by clinicians. They expressed (1) a desire for a prompt to redo stroke and bleeding risk assessments when needed and (2) a need for more education and awareness about the toolkit.

##### Desire for a Prompt to Redo Stroke and Bleeding Risk Assessments When Needed

All participants expressed that it would be helpful to have a reminder in the EMR system to prompt them to redo a stroke and bleeding risk assessment when certain CHADS-related patient characteristics (ie, age, new comorbidities) changed. A family physician described how receiving reminders would be helpful as long as it wasn’t too frequent:

I think [it would be helpful]...if it did it at the age 65 and 75, whenever the brackets are, not every birthday...Or if there was somehow when...they get a new diagnosis of something if it could prompt you to think of it that would be really helpful...

##### Need for More Education and Awareness About the Toolkit

Participants described the need to provide education and awareness about the toolkit and its functionalities to clinicians. A family physician suggested the need for training and hands-on practice with the toolkit to gain familiarity with it:

I think it does take some time to get familiar with using these so it’s like practice. So I think it would be best if people...had training or something...just some examples of case studies or something...you have a new patient with AFib, this is what you would do and then you would go here because for me to see this for the very first time this form to fill out I think would take a lot of time.

From survey results, 50% (5/10) of participants thought an orientation session was necessary to introduce the AF EMR toolkit. Most (64%, 7/11) participants believed an orientation session would increase usage of the toolkit.

#### Perceived Impact of the Toolkit on Patient Care and Workflow

##### Intention to Continue Using the EMR Toolkit for AF

All interviewed participants expressed their intent to continue using the toolkit to help guide AF management. A family physician acknowledged the benefits of certain aspects of the toolkit but emphasized her preference for the stroke and bleeding risk calculator:

I mean in all honesty I think if there was one thing that I for sure I’ll use is the stroke and bleeding risk piece of it. The initial assessment, I think I would continue to use just [so] I take an AFib history in a very organized way, but if I’m super busy and I kind of forget that one might be the first to go but definitely the stroke and bleeding risk I would in terms of guiding managing I definitely would use that.

From survey results, 82% (9/11) of surveyed clinicians intended to continue using aspects of the AF EMR toolkit in the future. Overall, 91% (10/11) of participants would recommend the EMR toolkit for AF to other clinicians. However, remembering to use the toolkit was suggested as the main barrier to its use.

##### Increased Thoroughness and Quality of AF-Related Care

Interviewed participants expressed that the toolkit prompted them to provide a more thorough assessment for patients with AF. A family physician described how the toolkit “prompted me to do things that I probably maybe wouldn’t have done.” This finding was supported by survey results with the majority (82%, 9/11) of participants agreeing that the toolkit increased the thoroughness of their assessments of patients as recommended by AF guidelines. Most (73%, 8/11) participants believed the toolkit increased the quality of AF-related care received by their patients.

## Discussion

### Principal Findings

The EMR toolkit for AF was designed as a resource for primary care clinicians to help guide decision making and improve the proportion of patients who are receiving guideline-concordant AF care. Through our interdisciplinary and human factors approach, we were able to develop a toolkit that incorporated perspectives from individuals with different areas of expertise—family medicine, pharmacy, cardiology, and nursing—and that was optimized for use in a large, busy family practice environment.

As a result of usability testing, we were able to develop a user-centered intervention that met the needs of primary care clinicians by identifying potential problems and areas for improvement early in the development stages. Nine iterations of the toolkit were created in response to feedback from clinicians who participated in Phase 1 of the study to make the toolkit more effective; interface-related changes were made, additional AF-related resources were added, and functionality issues were fixed. After these improvements to the toolkit were made, clinicians provided positive feedback regarding the toolkit and its perceived impact. Clinicians expressed that the toolkit improved accessibility to AF-related information and resources, served as a reminder for guideline-concordant AF management, and was easy to use. Most clinicians intended to continue using the toolkit for patient care, which may be attributable to its user-centered design. With respect to impact on care, clinicians believed the toolkit increased the thoroughness of their assessments for patients with AF and improved the quality of AF-related care received by their patients.

The results of this pilot study informed the refinement of the toolkit to make it a more effective, holistic, and user-centered intervention. The final version of the toolkit will be implemented across primary care clinics in Ontario in a cluster-randomized controlled trial examining its impact on guideline-concordant AF care. Through this mixed-methods study, we were able to demonstrate the utility of a human factors approach to piloting and refining an intervention prior to wide-scale implementation.

### Comparison With Prior Work

The importance of incorporating the end users’ needs and workflow into the design of information technology (IT) interventions has been well documented [16-20]. Additionally, research has found that the usability of a system is a major theme impacting use of IT interventions [21,22]. Incorporating these principles, participants expressed positive feedback about the EMR toolkit for AF and an intention to continuing using the toolkit for AF management. The technology acceptance model also suggests that the perceived usefulness and ease-of-use of a technology influences end users’ intentions to use the technology. Our study supports this theory. This study provides novel information on the utility of a human factors approach to the development of an IT intervention for AF in the primary care setting.

### Limitations

This study has several limitations. The study was conducted in urban-based, academic family health centers, the sample size for the survey was small, and the selection of clinicians may have been biased toward those who are accepting of novel quality improvement interventions. As such, the results may not be generalizable to all primary care practices in Ontario. The study also sought out self-reported experiences, and as a result, participant responses may be impacted by response bias and recall bias. However, data were collected through three methods—interviews, observations, and surveys—which provided similar results, reinforcing the themes identified.

### Conclusion

Although an experienced multidisciplinary team carefully designed the EMR toolkit, we found that the use of a human factors approach enabled the development of an intervention that better met the needs of primary care clinicians. The positive feedback surrounding the EMR toolkit for AF and its perceived impact on patient care can be attributed to the adoption of a user-centered design that merged clinically important information about AF management with user needs. This study demonstrates the utility of a human factors approach to piloting and refining an intervention prior to wide-scale implementation.

## Abbreviations

AF: atrial fibrillation
EMR: electronic medical record

## Appendix

Multimedia Appendix 1 EMR toolkit screenshot.

## References

[1] Wolf PA, Abbott RD, Kannel WB (1991). Atrial fibrillation as an independent risk factor for stroke: The Framingham Study. Stroke.

[2] (2014). Heart and Stroke Foundation.

[3] Gladstone DJ, Bui E, Fang J, Laupacis A, Lindsay MP, Tu JV, Silver FL, Kapral MK (2009). Potentially preventable strokes in high-risk patients with atrial fibrillation who are not adequately anticoagulated. Stroke.

[4] Skanes AC, Healey JS, Cairns JA, Dorian P, Gillis AM, McMurtry MS, Mitchell LB, Verma A, Nattel S, Canadian Cardiovascular Society Atrial Fibrillation Guidelines Committee (2012). Focused 2012 update of the Canadian Cardiovascular Society atrial fibrillation guidelines: Recommendations for stroke prevention and rate/rhythm control. Can J Cardiol.

[5] Louis KM, Martineau J, Rodrigues I, Fournier M, Berbiche D, Blais N, Ginsberg J, Blais L, Montigny M, Perreault S, Vanier M, Lalonde L (2010). Primary care practices and determinants of optimal anticoagulation management in a collaborative care model. Am Heart J.

[6] Peterson GM, Boom K, Jackson SL, Vial JH (2002). Doctors' beliefs on the use of antithrombotic therapy in atrial fibrillation: Identifying barriers to stroke prevention. Intern Med J.

[7] Bungard TJ, Ghali WA, McAlister FA, Buchan AM, Cave AJ, Hamilton PG, Mitchell LB, Shuaib A, Teo KK, Tsuyuki RT (2001). Physicians' perceptions of the benefits and risks of warfarin for patients with nonvalvular atrial fibrillation. CMAJ.

[8] Beyth RJ, Antani MR, Covinsky KE, Miller DG, Chren MM, Quinn LM, Landefeld CS (1996). Why isn't warfarin prescribed to patients with nonrheumatic atrial fibrillation?. J Gen Intern Med.

[9] Devereaux PJ, Anderson DR, Gardner MJ, Putnam W, Flowerdew GJ, Brownell BF, Nagpal S, Cox JL (2001). Differences between perspectives of physicians and patients on anticoagulation in patients with atrial fibrillation: Observational study. BMJ.

[10] Protheroe J, Fahey T, Montgomery AA, Peters TJ (2000). The impact of patients' preferences on the treatment of atrial fibrillation: Observational study of patient based decision analysis. BMJ.

[11] Bungard TJ, Ghali WA, McAlister FA, Buchan AM, Cave AJ, Hamilton PG, Mitchell LB, Shuaib A, Teo KK, Tsuyuki RT (2003). The relative importance of barriers to the prescription of warfarin for nonvalvular atrial fibrillation. Can J Cardiol.

[12] Chaudhry B, Wang J, Wu S, Maglione M, Mojica W, Roth E, Morton SC, Shekelle PG (2006). Systematic review: Impact of health information technology on quality, efficiency, and costs of medical care. Ann Intern Med.

[13] Hillestad R, Bigelow J, Bower A, Girosi F, Meili R, Scoville R, Taylor R (2005). Can electronic medical record systems transform health care? Potential health benefits, savings, and costs. Health Aff (Millwood).

[14] Adams WG, Mann AM, Bauchner H (2003). Use of an electronic medical record improves the quality of urban pediatric primary care. Pediatrics.

[15] Kohn L, Corrigan J, Donaldson M (1999). Institute of Medicine.

[16] Kjeldskov J, Skov MB, Stage J (2010). A longitudinal study of usability in health care: Does time heal?. Int J Med Inform.

[17] Lowry S, Ramaiah M, Patterson E, Brick D, Gurses A, Ozok A, Simmons D, Gibbons M (2014). National Institute of Standards and Technology, US Department of Commerce.

[18] Karsh B (2004). Beyond usability: Designing effective technology implementation systems to promote patient safety. Qual Saf Health Care.

[19] Rose AF, Schnipper JL, Park ER, Poon EG, Li Q, Middleton B (2005). Using qualitative studies to improve the usability of an EMR. J Biomed Inform.

[20] Johnson CM, Johnson TR, Zhang J (2005). A user-centered framework for redesigning health care interfaces. J Biomed Inform.

[21] Horsky J, Schiff GD, Johnston D, Mercincavage L, Bell D, Middleton B (2012). Interface design principles for usable decision support: A targeted review of best practices for clinical prescribing interventions. J Biomed Inform.

[22] Smelcer J, Miller-Jacobs H, Kantrovich L (2009). Usability of electronic medical records. J Usability Stud.

