# Supplementary material for: Evaluating the Usability and Perceived Impact of an Electronic Medical Record Toolkit for Atrial Fibrillation Management in Primary Care: A Mixed-Methods Study Incorporating Human Factors Design
Source: JMIR Hum Factors. 2016 Feb 17;3(1):e7. doi: 10.2196/humanfactors.4289 (PMC4811663; doi:10.2196/humanfactors.4289)
Supplement: Multimedia Appendix 1 [file humanfactors_v3i1e7_app1.pdf]

EMR Toolkit.

(A) Initial assessment form.

Feb 16, 2016 Atrial Fibrillation Initial Assessment AV

History

- AFIB confirmed via ECG
- document AFIB in CPP

Pattern

Severity of AFIB

learn more...

SUBJECTIVE

Current Symptoms:

- palpitations
- chest pain
- lightheadedness
- fatigue
- decrease in exercise capacity
- dyspnea
- syncope

Triggers:

(e.g. alcohol, caffeine)

Risk Factors

- Hypertension
- Sleep Apnea
- Diabetes
- Hyperthyroidism
- Left Ventricular Dysfunction
- Mitral Stenosis
- Rheumatic Heart Disease
- Coronary Heart Disease

OBJECTIVE

Apical HR:

- regular

BP:

- JVP elevated
- carotid bruit
- normal heart sounds
- murmur
- ankle edema
- peripheral pulses present

PLAN

Stroke Prevention

→ **calculate stroke risk**

- oral anticoagulation

more info

- ASA
- Other antiplatelet

Reassess stroke risk when:

1. Reached aged 65 yrs or 75 yrs
2. New dx of DM, CHF, HTN, stroke/TIA, vascular disease

Adherence

- adverse effects / bleeding

Rate Control

Target HR less than 100

- BB
- CCB
- digoxin

more info

Investigations

- TSH
- CBC
- ALT
- Hb A1C
- ECG
- echocardiogram
- sleep study
- stress test

Education

- Avoid alcohol, caffeine, nicotine
- Discussed medication side effects, interactions, and monitoring
- Patient handouts given

Follow-up

- Referral to cardiology
- Referral to internal medicine

Followup in:

Finish

CognisantMD

(B) Follow-up visit form.

Feb 16, 2016 AFIB Follow Up Visit AV

SUBJECTIVE

Current Symptoms:

- palpitations
- fatigue
- dyspnea
- lightheadedness
- chest pain
- bleeding
- bruising

Severity of AFIB

learn more...

OBJECTIVE:

Physical Exam:

Apical HR:

BP:

- Normal heart sounds
- Chest Clear

→ **view Flowsheet**

CognisantMD

Latest Labs:

Monitored by specialist:

Last monitored on: mmm d, yyyy

Hb	PRIVATE
Platelets	PRIVATE
INR	2.5
Creatinine	70
eGFR	PRIVATE

ASSESSMENT:

Current Stroke Prevention:

→ **calculate stroke risk**

- oral anticoagulation
- ASA
- Other antiplatelet

Reassess stroke risk if:

1. Reached aged 65 yrs or 75 yrs
2. New dx of DM, CHF, HTN, stroke/TIA, vascular disease

Adherence

Current Rate Control:

- CCB
- BB
- Digoxin

Other:

Adherence

PLAN

Rate/Rhythm

- Continue current regimen
- Change current treatment

more info

Stroke Prevention

- Continue current regimen
- Change current treatment

more info

Investigations

- CBC
- INR
- Cr / eGFR

Education

- Patient handouts given

Follow up in

Other:

Finish

(C) Stroke and bleeding risk calculator.

☐ **Special Note** Stroke and Bleeding Risk Tool JEL/dc

CognisantMD™ ⓧ

Stroke Risk

- ☐ Moderate to severe mitral stenosis

CHADS2

- ☐ Congestive heart failure or EF <= 35%
- ☑ Hypertension
- ☑ Age >= 75 Learn more
- ☐ Diabetes Mellitus
- ☐ Stroke, TIA or systemic emboli

- ☐ Vascular disease (previous MI, PAD or aortic plaque)
- ☐ Age 65 to 74 Learn more
- ☐ Female

- ☐ None of the above

Annual Stroke Risk: 4.0% (3.1-5.1)

Bleeding Risk

- ☐ Systolic >= 160mmHg
- ☐ Abnormal renal function (dialysis, transplant or Cr > 200)
- ☐ Abnormal liver function (cirrhosis or bili >2X normal + AST/ALT/ALP > 3X normal)
- ☐ Stroke
- ☐ Bleeding history or disposition Learn more
- ☐ Labile INR (INR in range less than 60% of time)
- ☑ Elderly (Age > 65 years)
- ☐ Drugs (concomitant NSAIDs, antiplatelets) Learn more
- ☐ Alcohol (more than 7 drinks / week)

- ☐ None of the above
- ☐ Other considerations (e.g. risk of frequent/traumatic falls) Learn more

Annual Bleeding Risk: 1.02%

Recommendations:

** Based on stroke risk, oral anticoagulation is recommended. **
Remind the patient that 20% of strokes due to AFIB are fatal and 60% result in permanent disability [1]. Treatment with anticoagulation reduces this risk by 60-70% and aspirin reduces this risk by 20-25% [2].

Latest recorded systolic BP:PRIVATE
Latest creatinine: 70

→ References
